# Pralidoxime in Acute Organophosphorus Insecticide Poisoning—A Randomised Controlled Trial

**DOI:** 10.1371/journal.pmed.1000104

**Published:** 2009-06-30

**Authors:** Michael Eddleston, Peter Eyer, Franz Worek, Edmund Juszczak, Nicola Alder, Fahim Mohamed, Lalith Senarathna, Ariyasena Hittarage, Shifa Azher, K. Jeganathan, Shaluka Jayamanne, Ludwig von Meyer, Andrew H. Dawson, Mohamed Hussain Rezvi Sheriff, Nick A. Buckley

**Affiliations:** 1 Centre for Tropical Medicine, Nuffield Department of Clinical Medicine, University of Oxford, United Kingdom; 2 Ox-Col Collaboration, Department of Clinical Medicine, Faculty of Medicine, University of Colombo, Sri Lanka; 3 South Asian Clinical Toxicology Research Collaboration, Sri Lanka; 4 Walther Straub Institute of Pharmacology and Toxicology, Ludwig Maximilians University, Munich, Germany; 5 Bundeswehr Institute of Pharmacology and Toxicology, Munich, Germany; 6 Centre for Statistics in Medicine, Wolfson College, University of Oxford, England; 7 Anuradhapura General Hospital, North Central Province, Sri Lanka; 8 Polonnaruwa General Hospital, North Central Province, Sri Lanka; 9 Institute of Legal Medicine, Ludwig Maximilians University, Munich, Germany; 10 School of Public Health, University of Newcastle, Australia; 11 Professorial Unit, Department of Medicine, University of New South Wales, Sydney, Australia; University College London, United Kingdom

## Abstract

**Background:**

Poisoning with organophosphorus (OP) insecticides is a major global public health problem, causing an estimated 200,000 deaths each year. Although the World Health Organization recommends use of pralidoxime, this antidote's effectiveness remains unclear. We aimed to determine whether the addition of pralidoxime chloride to atropine and supportive care offers benefit.

**Methods and Findings:**

We performed a double-blind randomised placebo-controlled trial of pralidoxime chloride (2 g loading dose over 20 min, followed by a constant infusion of 0.5 g/h for up to 7 d) versus saline in patients with organophosphorus insecticide self-poisoning. Mortality was the primary outcome; secondary outcomes included intubation, duration of intubation, and time to death. We measured baseline markers of exposure and pharmacodynamic markers of response to aid interpretation of clinical outcomes. Two hundred thirty-five patients were randomised to receive pralidoxime (121) or saline placebo (114). Pralidoxime produced substantial and moderate red cell acetylcholinesterase reactivation in patients poisoned by diethyl and dimethyl compounds, respectively. Mortality was nonsignificantly higher in patients receiving pralidoxime: 30/121 (24.8%) receiving pralidoxime died, compared with 18/114 (15.8%) receiving placebo (adjusted hazard ratio [HR] 1.69, 95% confidence interval [CI] 0.88–3.26, *p* = 0.12). Incorporating the baseline amount of acetylcholinesterase already aged and plasma OP concentration into the analysis increased the HR for patients receiving pralidoxime compared to placebo, further decreasing the likelihood that pralidoxime is beneficial. The need for intubation was similar in both groups (pralidoxime 26/121 [21.5%], placebo 24/114 [21.1%], adjusted HR 1.27 [95% CI 0.71–2.29]). To reduce confounding due to ingestion of different insecticides, we further analysed patients with confirmed chlorpyrifos or dimethoate poisoning alone, finding no evidence of benefit.

**Conclusions:**

Despite clear reactivation of red cell acetylcholinesterase in diethyl organophosphorus pesticide poisoned patients, we found no evidence that this regimen improves survival or reduces need for intubation in patients with organophosphorus insecticide poisoning. The reason for this failure to benefit patients was not apparent. Further studies of different dose regimens or different oximes are required.

**Trial Registration:**

Controlled-trials.com ISRCTN55264358

*Please see later in the article for Editors' Summary*

## Abstract 2

In a randomized controlled trial of individuals who had taken organophosphorus insecticides, Michael Eddleston and colleagues find that there is no evidence that the addition of the antidote pralidoxime offers benefit over atropine and supportive care.

## Editors' Summary

**Background:**

Each year, about 200,000 people worldwide die from poisoning with organophosphorous insecticides, toxic chemicals that are widely used in agriculture, particularly in developing countries. Organophosphates disrupt communication between the brain and the body in both insects and people. The brain controls the body by sending electrical impulses along nerve cells (neurons) to the body's muscle cells. At the end of the neurons, these impulses are converted into chemical messages (neurotransmitters), which cross the gap between neurons and muscle cells (the neuromuscular junction) and bind to proteins (receptors) on the muscle cells that pass on the brain's message. One important neurotransmitter is acetylcholine. This is used at neuromuscular junctions, in the part of the nervous system that controls breathing and other automatic vital functions, and in parts of the central nervous system. Normally, the enzyme acetylcholinesterase quickly breaks down acetylcholine after it has delivered its message, but organophosphates inhibit acetylcholinesterase and, as a result, disrupt the transmission of nerve impulses at nerve endings. Symptoms of organophosphate poisoning include excessive sweating, diarrhea, muscle weakness, and breathing problems. Most deaths from organophosphate poisoning are caused by respiratory failure.

**Why Was This Study Done?:**

Treatment for organophosphorous insecticide poisoning includes resuscitation and assistance with breathing (intubation) if necessary and the rapid administration of atropine. This antidote binds to “muscarinic” acetylcholine receptors and blocks the effects of acetylcholine at this type of receptor. Atropine can only reverse some of the effects of organophosphate poisoning, however, because it does not block the activity of acetylcholine at its other receptors. Consequently, the World Health Organization (WHO) recommends that a second type of antidote called an oxime acetylcholinesterase reactivator be given after atropine. But, although the beneficial effects of atropine are clear, controversy surrounds the role of oximes in treating organophosphate poisoning. There is even some evidence that the oxime pralidoxime can be harmful. In this study, the researchers try to resolve this controversy by studying the effects of pralidoxime treatment on patients poisoned by organophosphorous insecticides in Sri Lanka in a randomized controlled trial (a study in which groups of patients are randomly chosen to receive different treatments).

**What Did the Researchers Do and Find?:**

The researchers enrolled 235 adults who had been admitted to two Sri Lankan district hospitals with organophosphorous insecticide self-poisoning (in Sri Lanka, more than 70% of fatal suicide attempts are the result of pesticide poisoning). The patients, all of whom had been given atropine, were randomized to receive either the WHO recommended regimen of pralidoxime or saline. The researchers determined how much and which pesticide each patient had been exposed to, measured the levels of pralidoxime and acetylcholinesterase activity in the patients' blood, and monitored the patients' progress during their hospital stay. Overall, 48 patients died—30 of the 121 patients who received pralidoxime and 18 of the 114 control patients. After adjusting for the baseline characteristics of the two treatment groups and for intubation at baseline, pralidoxime treatment increased the patients' risk of dying by two-thirds, although this increased risk of death was not statistically significant. In other words, this result does not prove that pralidoxime treatment was bad for the patients in this trial. However, in further analyses that adjusted for the ingestion of different insecticides, the baseline levels of insecticides in patients' blood, and other prespecified variables, pralidoxime treatment always increased the patients' risk of death.

**What Do These Findings Mean?:**

These findings provide no evidence that the WHO recommended regimen of pralidoxime improves survival after organophosphorous pesticide poisoning even though other results from the trial show that the treatment reactivated acetylcholinesterase. Indeed, although limited by the small number of patients enrolled into this study (the trial recruited fewer patients than expected because results from another trial had a deleterious effect on recruitment), these findings actually suggest that pralidoxime treatment may be harmful at least in self-poisoned patients. This suspicion now needs be confirmed in trials that more fully assess the risks/benefits of oximes and that explore the effects of different dosing regimens and/or different oximes.

**Additional Information:**

Please access these Web sites via the online version of this summary at http://dx.doi.org/10.1371/journal.pmed.1000104.

- The US Environmental Protection Agency provides information about all aspects of insecticides (in English and Spanish)
- Toxtown, an interactive site from the US National Library of Medicine provides information on exposure to pesticides and other environmental health concerns (in English and Spanish)
- The US National Pesticide Information Center provides objective, science-based information about pesticides (in English and Spanish)
- MedlinePlus also provides links to information on pesticides (in English and Spanish)
- For more on Poisoning Prevention and Management see WHO's International Programme on Chemical Safety (IPCS)
- WikiTox, a clinical toxicology teaching resource project, has detailed information on organophosphates

## Introduction

Organophosphorus (OP) insecticide poisoning is a major global clinical problem, killing an estimated 200,000 people each year [1],[2]. Restricting agricultural use of highly toxic OP insecticides will reduce regional suicide rates [3],[4]. However, current agricultural policies [5] make it unlikely that they will soon be banned. Effective clinical therapies are required [6].

OP compounds inhibit acetylcholinesterase (EC 3.1.1.7), resulting in overstimulation of cholinergic synapses [7],[8]. Patients die mostly from respiratory failure and lung injury [8],[9], although there is variability in the clinical syndrome [10]–[12]. Treatment involves resuscitation, administration of the muscarinic antagonist atropine [13], and an oxime acetylcholinesterase reactivator [14], such as pralidoxime, and assisted ventilation as necessary [15]. The beneficial effects of atropine are clear [13],[16]. By contrast, the role of oximes is still the subject of much debate [17]–[19].

Clinical experience in Asia with regimens of 1 g pralidoxime every 4–6 h for 1–3 d has lead to widespread doubt about its efficacy in treatment of OP insecticide poisoning [20],[21]. Two meta-analyses concluded that pralidoxime causes harm [18],[22] but both included nonrandomised historical studies as well as randomised controlled trials (RCTs). Proponents of oximes, including the World Health Organization (WHO), believe that the doses used were too low to be effective and recommended a higher dosing regimen (at least 30 mg/kg pralidoxime salt loading dose followed by 8 mg/kg infusion) [23],[24]. Furthermore, the studies did not account for variable acetylcholinesterase ageing (a nonenzymatic alteration of phosphorylated acetylcholinesterase that prevents reactivation by oximes) caused by different classes of insecticide [14].

We set up an RCT in two Sri Lankan district hospitals in 2004 to compare the WHO-recommended regimen of pralidoxime with placebo in OP insecticide poisoning.

## Methods

The RCT was conducted in Anuradhapura and Polonnaruwa district hospitals, Sri Lanka. Ethics approval was received from the Faculty of Medicine Ethics Committee, Colombo, and Oxfordshire Clinical Research Ethics Committee. Written informed consent was taken from each patient, or their relative (for patients unconscious or under the age of 16 y), in their own language.

### Participants

We approached all patients with OP insecticide self-poisoning admitted to adult wards who required atropine according to our protocol [15]. The exclusion criteria were: age <14 y, known pregnancy, receipt of pralidoxime at a transferring hospital, and previous recruitment to this RCT. The OP insecticide was identified from the history or clinical syndrome (sources previously found to be highly accurate [11]). Patients were seen by study doctors within 30 min of admission and treated as described [15].

### Outcome, Objectives, and Hypotheses

The primary aim was to determine whether pralidoxime chloride reduced all-cause mortality during hospital admission after OP self-poisoning compared with no pralidoxime. Secondary outcomes included intubation, time to intubation, time ventilated, and time to death.

We performed prespecified subgroup analyses to determine whether any effect was consistent between patients poisoned with dimethyl versus diethyl organophosphorus insecticides, patients poisoned by the two most common pesticides (dimethoate, chlorpyrifos [11]), and whether any effect was dependent on time from ingestion to treatment or Glasgow coma scale (GCS) score on admission. We adjusted these analyses according to baseline red cell acetylcholinesterase (EC 3.1.1.7) ageing and plasma insecticide concentration measured retrospectively.

A post-hoc analysis was performed to assess whether any effect noted was consistent for patients intubated or not intubated at baseline.

### Randomisation

Patients were randomised into one of two study arms to receive saline placebo or pralidoxime chloride (2 g loading dose over 20 min, then a constant infusion of 0.5 g/h until a maximum of 7 d, atropine had not been required for 12–24 h, or death). The random allocation sequence was generated by computer and incorporated into a programme written for recruitment, randomisation, and event recording. Stratified block randomisation was performed using: (i) chemical structure (diethyl, dimethyl, unknown/other); (ii) reported time between poisoning and recruitment (<4 h; 4–12 h; >12 h; unknown); (iii) status on admission (GCS 14–15/15, GCS <14), and (iv) allocation in a concurrent RCT of activated charcoal [25].

The allocation sequences were generated independently by the statistician and implemented by the programmer, neither of whom interacted with patients. Variable block sizes were used to allocate patients in equal numbers to each treatment group using Stata v. 7 software (ralloc subroutine version 3.2.5).

Participants were recruited and randomised by a study doctor at the bedside using a dedicated handheld computer at each study hospital. Randomisation occurred after baseline data had been entered, and could not be altered by study doctors. The recruiting doctor could not predict allocation accurately before randomisation.

### Study Drug

Pralidoxime chloride was supplied by Pharmalab (New South Wales, Australia) as a 6.25 g/250 ml preparation. The quality of each batch was checked independently (SGS Lanka Laboratories) by HPLC on arrival in Sri Lanka (pralidoxime detected and present at 92.5% to 110% of the expected quantity and pH 3.5 to 4.5 [USP standards]). All batches used for the study fulfilled USP standards.

The study was double-blind. The pralidoxime and placebo were provided in batches of vials, identical except for a serial number starting with one of two letters: A or B, C or D, etc. At randomisation, the computer program specified a letter; vials with that letter were used for that patient. At intervals, the letter pairs were shifted to the next pair to reduce the risk of unblinding. Blood samples were subsequently assayed for pralidoxime; this showed that all patients received the correct allocation.

### Blood Sampling and Analysis of Pharmacokinetics and Pharmacodynamics

Blood samples were taken from patients on recruitment and at intervals thereafter for assay of plasma butyrylcholinesterase and red cell acetylcholinesterase activity [26], pralidoxime, and insecticide concentration. Sampling and assays were carried out as described [11],[27]. A technical problem caused the analysis of samples from 30% of patients to be delayed, allowing ageing of inhibited acetylcholinesterase to continue during storage (resulting in a 200 mU/µmol Hb reduced maximal reactivation of diethyl-inhibited acetylcholinesterase but little apparent difference for dimethyl-inhibited enzyme). These samples were included in the results and reduced the overall median acetylcholinesterase reactivation ex-vivo.

### Sample Size

We calculated that to detect whether pralidoxime reduced the case fatality in symptomatic patients from 25% to 19% (two-sided significance level of 5%, power 80%), a minimum of 750 patients was required in each arm. The trial was set up as a superiority trial.

### Independent Data Monitoring Committee

An independent data monitoring committee (IDMC) was established for this and the concurrent trial [25]. Interim analyses were to be supplied by the trial statistician to the IDMC Chair as often as requested. In the light of interim data, and emerging evidence from other studies, the IDMC then informed the principal investigator if in their view there was proof beyond reasonable doubt that the data indicated that any part of the protocol under investigation became clearly indicated or contraindicated, or it was evident that no clear outcome would be obtained. The trial stopped after the first interim analysis due to lack of recruitment.

### Statistical Analysis

Demographic factors and clinical characteristics were summarised with counts (percentages) for categorical variables and median (interquartile range [IQR]) for continuous variables, as none were expected to be normally distributed. The main analysis was carried out on an intention-to-treat basis. For the primary outcome, death, and for secondary outcome postrandomisation intubations, we reported the number and proportion of patients experiencing an event.

For outcomes where time-to-event was recorded, we used the logrank test to compare the treatment groups, producing Kaplan-Meier curves to illustrate the comparison. In addition, we calculated incidence rates and performed Cox's regression to estimate hazard ratios (HRs) (plus 95% confidence interval [CI] and *p*-values) to establish the magnitude and direction of the treatment effect, adjusted for stratification factors, hospital, and intubation at baseline.

It was unclear how GCS on admission and time since ingestion should be fitted in statistical models, so various models were fitted using different approaches. The optimal statistical model was chosen based on the lowest value for Akaike's information criterion (AIC) [28], a measure of the goodness of fit of a statistical model, as long as the model was stable (AIC penalizes more complex models).

The statistical test of interaction was used to examine whether the treatment effects were consistent across poison subgroups (dimethyl, diethyl, unknown) and in those intubated/not intubated at baseline. A term representing the interaction was entered into the baseline statistical model and a Wald test performed to test for the presence of an interaction. Of note, however, the study size meant that we had limited power for analyzing interactions. An exploratory analysis using Cox's regression investigated the effects of potentially important prognostic factors such as percentage of aged acetylcholinesterase on admission and OP concentration on admission.

The Chi-squared test was used to compare the proportions of patients dying in red cell acetylcholinesterase activity groups. Median red cell acetylcholinesterase activity in survivors and fatalities, and median length of time intubated in each group, were compared using the Mann-Whitney U test.

## Results

Patients were enrolled from 26 May 2004 until 18 October 2006. Unfortunately, discussion of the results of an RCT [29] performed in Baramati, India, that suggested marked benefit from pralidoxime at a seminar in August 2005, resulted in loss of equipoise (the perception of treatments being of equal value) by clinicians, a fall off in recruitment, and early termination of the trial.

### Participants

**Figure 1:**
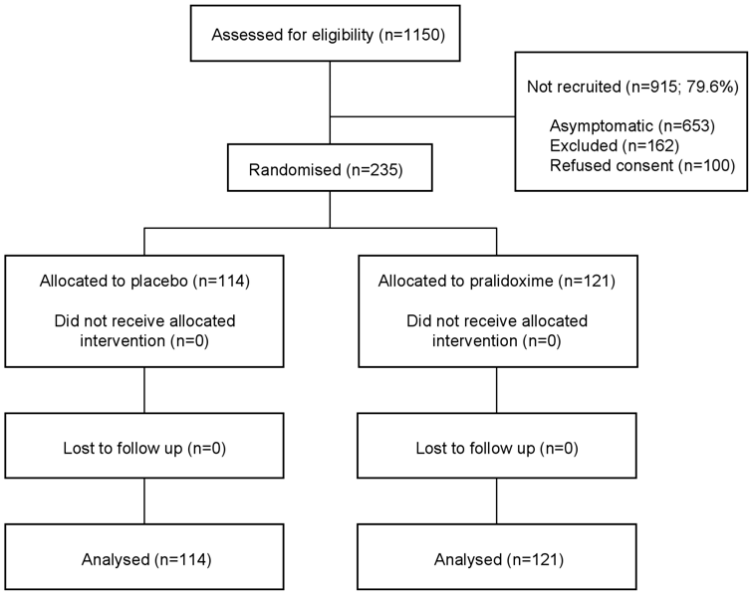
CONSORT flow diagram of progress through the RCT. 162 patients were excluded due to receiving pralidoxime in the referring hospital (151), being pregnant (7), or being less than 14 y old (4).

A total of 1,150 patients with OP poisoning were assessed on admission; 653 were asymptomatic, 162 excluded for other reasons, and 100 refused consent (Figure 1). 235 symptomatic patients were eligible, consented, and randomised into the trial: 114 received saline placebo and 121 received pralidoxime chloride.

Baseline demographic and clinical characteristics are presented in Tables 1 and 2. Although broadly similar, there were differences due to the relatively small number of patients recruited. In particular, more severely poisoned patients were allocated to pralidoxime, as shown by the proportion who were intubated before randomisation and had a GCS <14/15 (Table 1). Median time to recruitment was 4.3 h (IQR 2.9–7.6 h) postingestion.

**Table 1:** Baseline demographic and clinical characteristics.

Baseline Characteristic	Subcategory	Placebo (*n* = 114)	Pralidoxime (*n* = 121)
**Age, y, median (IQR)**		29.5 (23 to 42)	31 (22 to 48)
**Males, ** ***n*** ** (%)**		92 (80.7)	96 (79.3)
**Systolic BP, mmHg, mean (SD)**		116 (19.8)	118 (22.7)
**Diastolic BP, mmHg, mean (SD)**		76 (13.4)	76 (17.2)
**Pulse mean, bpm, mean (SD)**		101 (22)	97 (21)
**Time since ingestion, h, median (IQR)**		4.4 (2.9 to 7.4); *n* = 112	4.3 (2.9 to 7.8); *n* = 116
**Time since ingestion using categorical variable derived from times provided, ** ***n*** ** (%)**	<4 h	53 (46.5)	51 (42.2)
	4–12 h	41 (36.0)	45 (37.2)
	>12 h	18 (15.8)	20 (16.5)
	Unknown	2 (1.8)	5 (4.1)
**Charcoal allocation for those in RCT, ** ***n*** ** (%)**	Multiple dose activated charcoal	8 (7.0)	13 (10.7)
	Single dose activated charcoal	11 (9.7)	14 (11.6)
	No activated charcoal	10 (8.8)	13 (10.7)
	Not in RCT	85 (74.6)	81 (66.9)
**Charcoal treatment, ** ***n*** ** (%)**	Multiple dose activated charcoal	8 (7.0)	13 (10.7)
	Single dose activated charcoal	31 (27.2)	42 (34.7)
	No activated charcoal	75 (65.8)	66 (54.6)
**GCS score, median (IQR)**		15 (12 to 15)	14 (10 to 15)
**GCS score on admission, ** ***n*** ** (%)**	GCS 14 or 15	79 (69.3)	73 (60.3)
	GCS<14	35 (30.7)	48 (39.7)
	GCS 11–13	15 (13.2)	16 (13.2)
	GCS 7–10	5 (4.4)	6 (5.0)
	GCS 3–6	15 (13.2)	26 (21.5)
**Intubated at baseline, ** ***n*** ** (%)**		16 (14.0)	24 (19.8)

**Table 2:** Baseline analytical laboratory characteristics.

Baseline Characteristics	Subcategory	Placebo (*n* = 114)	Pralidoxime (*n* = 121)
**OP insecticide class at randomisation, ** ***n*** ** (%)**	Dimethyl	47 (41.2)	46 (38.3)
	Diethyl	49 (43.0)	54 (45.0)
	Unknown	18 (15.8)	20 (16.7)
**OP insecticide class after lab analysis, ** ***n*** ** (%)**	Number	112	121
	Dimethyl	33 (29.5)	39 (32.2)
	Diethyl	50 (44.6)	62 (51.2)
	S-alkyl	2 (1.8)	0
	Mixed	2 (1.8)	1 (0.8)
	Unknown	21 (18.8)	16 (13.2)
	No OP detected	4 (3.6)	3 (2.5)
**BuChE activity on admission, mU/ml**	Number	103	106
	Median (IQR)	110 (9 to 746)	86 (6 to 920)
	Dimethyl, median (IQR) (*n*)	431 (20 to 1606) (*n* = 41)	733 (73 to 1876) (*n* = 39)
	Diethyl, median (IQR) (*n*)	15 (0 to 144) (*n* = 46)	10 (0 to 99) (*n* = 49)
	Other or unknown, median (IQR) (*n*)	122 (34 to 818) (*n* = 16)	121 (10 to 740) (*n* = 17)
**Red cell AChE activity before treatment, mU/µmol Hb**	Number	92	102
	Median (IQR)	28 (7 to 59)	44 (12 to 97)
	Dimethyl, median (IQR) (*n*)	9 (2 to 32) (*n* = 36)	17 (6 to 70) (*n* = 37)
	Diethyl, median (IQR) (*n*)	47 (27 to 65) (*n* = 40)	60 (34 to 116) (*n* = 47)
	Other or unknown, median (IQR) (*n*)	20 (6 to 115) (*n* = 16)	33 (4 to 68) (*n* = 17)
**Aged red cell AChE before Rx, %**	Number	92	101
	Median (IQR)	59 (34 to 96)	46 (29 to 89)
	Dimethyl, median (IQR) (*n*)	97 (61 to 100) (*n* = 36)	89 (56 to 99) (*n* = 36)
	Diethyl, median (IQR) (*n*)	34 (20 to 45) (*n* = 40)	35 (21 to 45) (*n* = 48)
	Other or unknown, median (IQR) (*n*)	84 (47 to 92) (*n* = 16)	72 (34 to 100) (*n* = 16)

Data were collected on admission to hospital; recruitment occurred soon after.

Abbreviations: AChE, acetylcholinesterase; BuChE, butyrylcholinesterase.

### Assessment of Adequacy of the Pralidoxime Regimen

**Figure 2:**
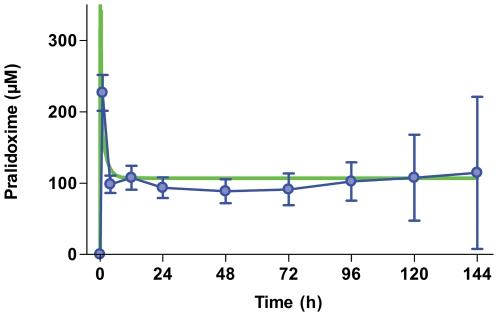
Pharmacodynamics of oxime administration. Time course of plasma pralidoxime concentration in patients allocated to receive pralidoxime chloride 2 g loading dose over 20 min followed by 0.5 mg/h until 7 d or until atropine no longer required (blue line, mean±SD; *n*≤85). A predicted time course (green line) was calculated for a 50 kg person using the kinetic data of Sidell and colleagues [42].

We first assessed the regimen's pharmacokinetics/dynamics to ensure that it had been adequate. It produced a steady state plasma pralidoxime concentration of approximately 100 µmol/l (Figure 2). We found no consistent difference in steady state concentration between patients who died and survivors (unpublished data).

**Figure 3:**
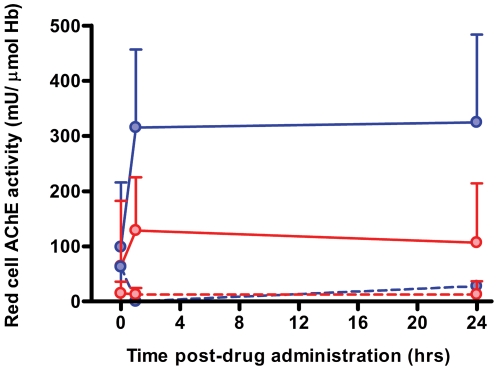
Pharmacokinetics of oxime administration. Red cell acetylcholinesterase activity (mean±SD) in patients poisoned by diethyl (blue) and dimethyl (red) OP insecticides, with (solid) and without (broken) pralidoxime chloride. Normal acetylcholinesterase activity is 600–700 mU/µmol Hb; an activity greater than 20%–30% of normal allows normal NMJ function [32]. Acetylcholinesterase was effectively reactivated after poisoning with diethyl insecticides but less so after dimethyl insecticide poisoning.

Pralidoxime effectively reactivated red cell acetylcholinesterase inhibited by diethyl OP insecticides but only moderately reactivated dimethyl OP-inhibited enzyme (Figure 3). Diethyl OP insecticides in this study included chlorpyrifos, quinalphos, and diazinon; dimethyl OP insecticides included dimethoate, fenthion, phenthoate, and oxydemeton-methyl. All are WHO Class II toxicity pesticides [30]. There was no reactivation for either class after placebo.

### Primary Outcome—Mortality

Overall mortality in the trial was 48/235 (20.4%). Case fatality was higher in patients receiving pralidoxime compared to placebo (30/121 [24.8%] versus 18/114 [15.8%]; crude estimated HR: 1.82 [95% CI 1.01–3.28, *p* = 0.05]). Adjustment for stratification variables, and for intubation at baseline, resulted in a revised estimated HR of 1.69 (95% CI 0.88–3.26, *p* = 0.12), suggesting no difference between groups.

**Figure 4:**
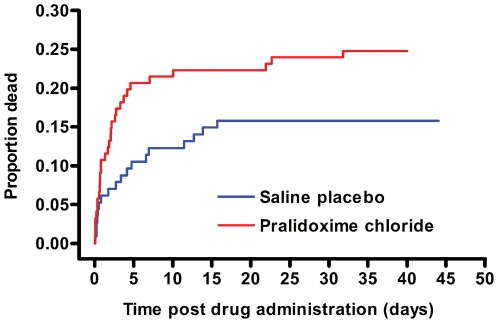
Timing of deaths in the two study arms. Cumulative percentage of patients who died. For the purposes of survival analysis, the clock has been started at randomisation and stops either at death or discharge (assumed to be 40 d if discharged alive sooner than 40 d).

**Figure 5:**
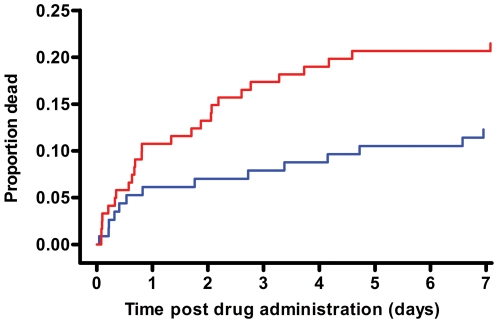
Timing of deaths during the first 6 d. For the purposes of survival analysis, the clock has been started at randomisation and stops either at death or discharge (assumed to be 40 d if discharged alive sooner than 40 d).

Patients died sooner after pralidoxime compared to placebo (Figure 4); however, this is partly explained by the baseline imbalance in GCS score (GCS<14/15: pralidoxime 48/121 [39.7%] versus control 35/114 [30.7%]; GCS<7/15: pralidoxime 26/121 [21.5%] versus control 15/114 [13.2%]; low GCS being a marker of poor prognosis [31]). Most of the difference in mortality occurred between 12 h and 5 d postrandomisation (Figure 5).

### Prespecified Subgroup Analysis

**Figure 6:**
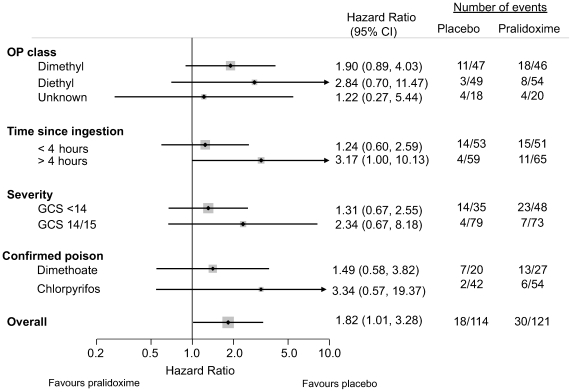
Forest plots of mortality for pralidoxime versus placebo for a priori defined study groups. The relatively few events precluded plots of adjusted analyses.

No differential effect was found for any of the prespecified subgroups; in particular there was no improvement in mortality for diethyl compounds (HR 2.84, 95% CI 0.70–11.47; Figure 6), despite good acetylcholinesterase reactivation.

We measured the plasma concentration of insecticide on admission, as well as the percentage of acetylcholinesterase that was aged at baseline, since both should affect the efficacy of pralidoxime [14]. All the information was present for 164 of 235 (69.8%) patients; the groups were similar at baseline except for small differences in OP class ingested and proportion intubated. The adjusted HR for death for this smaller group of 164 patients was 2.85 (95% CI 1.01–8.10, *p* = 0.05, AIC 183.9). Incorporating the baseline amount of acetylcholinesterase already aged and plasma OP concentration into the analysis increased the HR to 3.94 (1.25–12.36, *p* = 0.02) for patients receiving pralidoxime compared to placebo, further decreasing the likelihood that pralidoxime is beneficial.

We also examined the effect of pralidoxime for poisoning with the two most common insecticides (chlorpyrifos and dimethoate, Figure 6). Analysing patients poisoned by individual OPs reduces the confounding caused by the marked variability that exists between compounds of the same class. This analysis provided no evidence that pralidoxime offers benefit for either compound.

### Effectiveness of Pralidoxime in Reactivating Acetylcholinesterase in Fatal Cases

We analysed whether death occurred after effective acetylcholinesterase reactivation, using an activity of >199 mU/µmol Hb (20%–30% of normal) as an approximate level likely to be compatible with normal synaptic function [32]. In patients receiving pralidoxime, case fatality was lower in those with red cell acetylcholinesterase activity >199 mU/µmol Hb at 1 h and 24 hr, compared to those with activity <100 mU/µmol Hb (1 h: >199, case fatality: 6/62 [9.7%] versus <100, 15/30 [50.0%]; *p*<0.0001 Chi squared test; 24 h: >199, 5/51 [9.8%] versus <100, 7/24 [29.2%]; *p* = 0.03 Chi squared test). No such difference was seen in patients receiving placebo at 1 h (>199, 1/5 [20.0%] versus <100, 14/87 [16.1%]; *p* = 0.82 Chi squared test) and 24 h (>199, 0/4 [0%] versus <100, 9/71 [12.7%]; *p* = 0.45 Chi squared test). However, surprisingly, these data showed that survival was still high (84%–87%) in patients receiving placebo whose acetylcholinesterase activity remained very low at 1 and 24 h.

There was a significant difference in median post-treatment red cell acetyl-cholinesterase activity between survivors and fatalities in both arms (Table 3). The median acetylcholinesterase was lower in survivors who received placebo than in those who died after receiving pralidoxime.

**Table 3:** Median red cell acetylcholinesterase activity (mU/µmol Hb) in patients surviving or dying, by study arm, at 1 and 24 h post-treatment.

Time Point	Characteristic	Placebo Arm	Pralidoxime Arm
**Baseline, median (IQR)**		28 (7 to 59)	44 (12 to 97)
**1 h**	*n*	101	103
	Dead, median (IQR)	6 (0 to 15)	40 (21 to 206)
	Alive, median (IQR)	31 (12 to 65)	286 (147 to 400)
	Difference, median (95% CI; *p*-value)	23 (12 to 34; *p* = 0.0003)	182 (97 to 249; *p* = 0.0001)
**24 h**	*n*	86	86
	Dead, median (IQR)	2 (0 to 8)	62 (0 to 287)
	Alive, median (IQR)	45 (12 to 84)	302 (115 to 407)
	Difference, median (95% CI; *p*-value)	40 (18 to 53; *p* = 0.002)	135 (27 to 251; *p* = 0.01)

This table shows that patients who were allocated pralidoxime and survived had substantially higher red cell acetylcholinesterase activity after treatment than patients receiving pralidoxime who died. Patients who survived without receiving pralidoxime had only a marginally higher acetylcholinesterase activity post-treatment than people who died. This indicates that reactivated red cell acetylcholinesterase may not be essential for survival. Normal mean (±SD) red cell acetylcholinesterase in the laboratory was 651±18 mU/µmol Hb [27].

Only two of the 30 deaths in the pralidoxime arm occurred after the drug infusion was stopped in patients poisoned by fat-soluble OPs, with subsequent reinhibition of red cell acetylcholinesterase. This suggests that an inadequate duration of pralidoxime therapy was not the cause of the majority of deaths.

### Intubation

Eighty-six patients (86/235, 36.6%) required intubation. Forty (40/235, 17.0%) were intubated at baseline (Table 1), while 50 were intubated postrandomisation (50/235, 21.3%; four for a second time after postrandomisation extubation). Similar numbers of patients were intubated postrandomisation in each arm: 26/121 (21.5%) receiving pralidoxime and 24/114 (21.1%) receiving placebo (crude HR 1.23 [95% CI 0.70–2.14, *p* = 0.47], adjusted 1.25 [0.68–2.27, *p* = 0.47]). Incorporating baseline percentage aged acetylcholinesterase and plasma insecticide concentration into the statistical model increased the estimated HR to 1.80 (0.83–3.88, *p* = 0.14).

**Figure 7:**
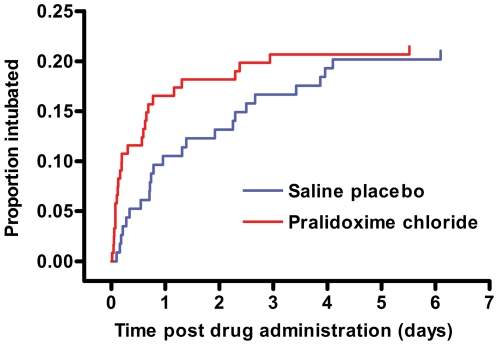
Timing of endotracheal intubation in the two study arms. Cumulative percentage intubated postrandomisation during the first 7 d. For the purposes of survival analysis, the clock has been started at randomisation, or in the case of those who were intubated at randomisation, when the patient was first extubated. The clock stops either at the first postrandomisation intubation, or at death or discharge (assumed to be 40 d if discharged alive sooner than 40 d).

Intubation occurred earlier in the pralidoxime arm (Figure 7). Patients receiving pralidoxime were intubated for shorter periods: median period 2.1 d (95% CI 0.8–4.8; *n* = 45) versus 6.5 d (1.8–10.1; *n* = 37; *p* = 0.02, Mann Whitney test). The picture was similar when we analysed only postrandomisation intubations: median period 3.5 d (0.8–4.7; *n* = 26) versus 8.0 d (4.4–10.2; *n* = 23; *p*≤0.001 Mann Whitney test). Some of this difference is likely to be due to the greater number of deaths among intubated patients treated with pralidoxime (25/48 [52.1%]) than those receiving placebo (15/38 [39.5%]).

**Figure 8:**
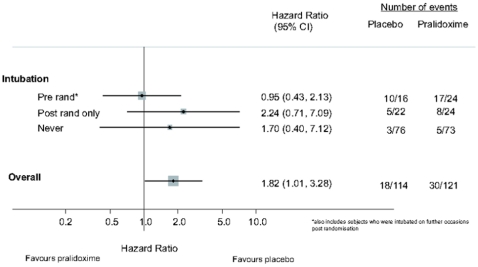
Forest plots of mortality for pralidoxime versus placebo for exploratory study subgroups. The relatively few events precluded plots of adjusted analyses.

A post-hoc exploratory analysis suggested that patients who received pralidoxime before intubation appeared to do worse than patients who received it after intubation at baseline (Figure 8).

### Adverse Events

Patients were assessed at the end of the loading dose and at 12 h intervals for adverse effects [33]. Tachycardia, hypertension (particularly diastolic), and vomiting were more common in patients receiving pralidoxime (Table 4). Over the next 72 h, only tachycardia and hypertension were more common in these patients.

**Table 4:** Adverse effects reported in each arm after the pralidoxime chloride/placebo loading dose or during the first 3 d of the constant infusion.

Adverse Effect	Loading dose (t = 20 min)			Constant infusion (t = 20 min to 72 h)		
	Placebo	Pralidoxime	*p*-Value	Placebo	Pralidoxime	*p*-Value
**Tachycardia** a **, ** ***n*** ** (%)**	30/110 (27.3)	61/115 (53.0)	<0.0001	54/111 (48.6)	85/114 (74.6)	<0.0001
**Hypertension** b **, ** ***n*** ** (%)**	2/110 (1.8)	27/115 (23.5)	<0.0001	16/111 (14.4)	34/114 (29.8)	0.005
**Headache, ** ***n*** ** (%)**	5/110 (4.5)	7/115 (6.1)	0.61	33/111 (29.7)	36/114 (31.6)	0.76
**Blurred vision, ** ***n*** ** (%)**	3/110 (2.7)	8/115 (7.0)	0.14	26/111 (23.4)	40/114 (35.1)	0.06
**Dizziness, ** ***n*** ** (%)**	8/110 (7.3)	9/115 (7.8)	0.88	35/111 (31.5)	31/114 (27.2)	0.48
**Nausea, ** ***n*** ** (%)**	12/110 (10.9)	13/115 (11.3)	0.93	33/111 (29.7)	25/114 (21.9)	0.18
**Vomiting, ** ***n*** ** (%)**	8/110 (7.3)	21/115 (18.3)	0.01	24/111 (21.6)	26/114 (22.8)	0.83
**Systolic BP, mmHg, mean (SD)**	116 (15.3)	129 (27.6)	<0.0001	ND	ND	
**Diastolic BP, mmHg, mean (SD)**	74 (13.5)	80 (20.2)	0.015	ND	ND	
**Pulse, bpm, mean (SD)**	99 (20.1)	113 (22.8)	<0.0001	ND	ND	

a Tachycardia, HR>100 bpm.

b Hypertension, systolic BP>159 and/or diastolic>99 mmHg.

ND, not done.

## Discussion

This trial showed no benefit from the administration of the WHO's recommended regimen of pralidoxime chloride to patients with symptomatic OP insecticide poisoning. The primary outcome—the (adjusted) HR showing higher mortality (1.69, 95% CI 0.88–3.26, *p* = 0.12) in patients receiving pralidoxime—is consistent with a broad range of effects: from a 12% reduction in mortality to a greater than 3-fold increase in mortality. However, the best estimate, i.e., the most likely effect from this trial, is a 69% increase in mortality due to the treatment. The results from other important outcomes in our trial, e.g. intubation, reinforce the finding of a lack of benefit for the treatment.

No subgroup appeared to derive differential benefit. All preplanned analyses failed to show that pralidoxime was beneficial, even for compounds such as chlorpyrifos for which there was expected to be the greatest chance of benefit and good reactivation of red cell acetylcholinesterase. Adjustment for the crucial baseline markers (OP ingested and extent of acetylcholinesterase ageing, measured for the first time in an OP trial) resulted in even less favorable estimates of effect. Our study shows that the WHO-recommended dose of pralidoxime is most likely to be ineffective, and may be harmful.

Any serious adverse effects occurring from pralidoxime were not clinically apparent. Case reports have suggested that pralidoxime causes cardiac dysrhythmias or respiratory arrest [18],[34] but these effects may also be induced by the OP insecticides. We noted marked diastolic hypertension in some patients receiving pralidoxime but no increased incidence of respiratory or cardiac arrest during or soon after the infusion started when the plasma concentration was at its greatest. We observed no other substantial adverse reactions that were attributed to pralidoxime at the time. However, there was a trend toward worse outcomes in patients not intubated before pralidoxime administration, suggesting that intubation may be protective against adverse effects (e.g., respiratory, arrest).

Three medium-sized RCTs of pralidoxime have previously been performed, two with pralidoxime chloride in Vellore [35],[36] and one with pralidoxime iodide in Baramati [29]. The studies compared different doses of these pralidoxime salts (Table 5). The Vellore studies compared a low-dose infusion with a single bolus [35] or placebo [36]. They found the low-dose infusion to be harmful, but there was a long delay to treatment in this trial and few patients could have benefited [17]. In contrast, the Baramati RCT found high doses (1 g of iodide salt, or 0.52 g of active pralidoxime cation, per hour) for the first 48 h after a loading dose to be beneficial, a difference previously related to more effective pralidoxime concentration, less ill patients, and very early treatment [37]. However, none of these studies took baseline measures of acetylcholinesterase ageing and inhibition or identified the responsible OP to allow adjustment for baseline differences. When we incorporated this information from our trial into our analysis, we found a decreased likelihood that pralidoxime is beneficial.

**Table 5:** Published RCTs of pralidoxime with more than 20 patients showing doses of the pralidoxime cation administered in each arm.

Trial	Salt^a^	Pralidoxime Cation per Gram of Salt	Arm 1 Cation Dose	Arm 2 Cation Dose
**Vellore ** [**35**]	Chloride^b^	0.795 g	0.80 g loading dose over 1–5 min	No loading dose, then infusion of 4.8 g over 1st 24 h, 2.4 g over 2nd 24 h, 1.6 g over 3rd 24 h, and 0.8 g over 4th 24 h
**Vellore ** [**36**]	Chloride^b^	0.795 g	None	No loading dose, then infusion of 9.5 g over 3 d^c^
**Baramati ** [**29**]	Iodide	0.520 g	1.04 g loading dose over 30 min, then 0.52 infused over 1 hr every 4 h	1.04 g loading dose over 30 min, then 0.52 g/h constant infusion for 48 h, then 0.52 g infused over 1 h every 4 h
**This trial**	Chloride	0.795 g	None	1.6 g loading dose over 20 min, then 0.4 g/h constant infusion for up to 7 d

a The different salts contain different quantities of pralidoxime [37],[43].

b Not stated in papers. Personal communication, Dr. J. V. Peter.

c Exact dosage regimen over the 3 d not stated in paper.

The Baramati RCT and our study used pralidoxime regimens (Table 5) similar to that recommended by the WHO [24] yet found very different results. It seems unlikely that this difference is due to the different salts since the chloride should be at least as safe as the iodide [37]. The median time to presentation in the two studies was not markedly different: 2 h versus 4.4 h. One obvious difference is the extent of supportive care. Baramati cases were treated in an intensive care unit and 66% were intubated at baseline, compared to 17.4% in our study, despite being less severely ill. While some hospitals in rural Asia are able to offer such a high standard of care, they are not the norm and most patients present to hospitals similar to our study sites in Sri Lanka. A second obvious difference is that high doses were used for only 48 h in the Baramati study but for up to 7 d in our study. The difference in mortality in our study continued to increase over time until at least 6 d postrandomisation (Figures 4 and 5).

This trial overlapped in part with another RCT of activated charcoal [25]. However, as shown in Table 1, only 69/235 (29.4%) patients were recruited into the charcoal RCT and their allocation was incorporated into the adjusted analysis. Furthermore, no effect of charcoal was noted in the RCT [25]. We therefore do not think that the charcoal RCT confounded the analysis of this RCT.

One limitation of this study was the lack of facilities for monitoring of patients that might have allowed us to better describe the cause of death in each patient, whether due to complications of prehospital aspiration or respiratory arrest, cholinergic syndrome, or cardiorespiratory arrest independent of the above that would suggest direct adverse effects of the pralidoxime. The study was therefore unable to explain why no benefit was found from this dose of pralidoxime; however, such information would not alter its conclusion.

A second limitation is that it was stopped early as a consequence of a loss of equipoise in recruiting clinicians after we became aware of the Baramati results. However, it has unique strengths, in particular baseline stratification of patients by insecticide and red cell acetylcholinesterase activity and ageing, as recommended by others [38]. Furthermore, despite falling short of our recruitment target, the clinical information we gathered, interpreted with the surrogate biochemical data, suggests that this regimen of pralidoxime is unlikely to be beneficial in our patient population.

Further interpretation of our results is not straightforward. We are faced with the perplexing fact that pralidoxime effectively reactivated diethyl-OP inhibited red cell acetylcholinesterase, but did not improve outcome. Might OPs have other detrimental effects that are not amenable to pralidoxime? The majority of the insecticides ingested were generic products formulated with xylene. It is possible that coformulants are responsible for a significant component of toxicity [9].

The evidence for pralidoxime effectiveness beyond the contradictory clinical trials is limited. Some evidence of effectiveness is claimed from animal studies, although species differences in acetylcholinesterase structure greatly affect OP binding and reversal by oximes [39]. Moreover, these studies are largely limited to single doses of pralidoxime given at the same time as a smallish dose of OP insecticide, in the absence of any standard titrated atropine treatment or supportive care [40]. They provide no support that continuous pralidoxime infusions in addition to usual care are useful. These studies do suggest we should move toward using oximes that are more effective than pralidoxime or have a better risk/benefit ratio [14],[40].

A second possible explanation is that pralidoxime is worthwhile but the dose too high. Pralidoxime has a high in vitro effect on human acetylcholinesterase at around 100 µmol/l [14], the target concentration of our regimen and the basis for the regimen being promoted by a WHO working group [24]. However, this may not necessarily be the optimal human dose in terms of risk/benefit. Further studies will be required to identify such a dose.

Another argument for a lower dose is that lesser degrees of reactivation may still be clinically useful. We have shown that red cell acetylcholinesterase activity in many survivors was less than 25% of normal, indicating that complete reactivation may be unnecessary. Aiming to achieve concentrations that achieve nearly full reactivation may lead to significant adverse effects.

The third possible explanation to consider is that there was a benefit in some patients but too many patients derived no benefit; that a more selective use might be useful. We chose, on pragmatic grounds, to administer pralidoxime for a maximum of 7 d, presuming that this would be the maximum period of active acetylcholinesterase inhibition in most patients. Oxime administration was stopped when patients no longer required atropine, indicating the presence of sufficient active acetylcholinesterase at muscarinic synapses.

However, retrospective analysis of red cell acetylcholinesterase activity indicates that many patients received pralidoxime at a time when no benefit was likely. This on its own does not provide an explanation for the adverse trend but could have been a contributing factor by increasing the time period for adverse effects from pralidoxime to manifest. Discontinuation or dose adjustment in response to rapid testing of the response to pralidoxime might have improved the overall risk/benefit ratio.

### Conclusion

Clinicians are now faced with a difficult situation. Should pralidoxime be given to patients with OP insecticide poisoning? Patients with relatively low-dose occupational poisoning by diethyl organophosphorus insecticides have been shown to clinically improve after low-dose pralidoxime administration [41]. However, for self-poisoned patients, we have no consistent clinical trial evidence for the use of this regimen of pralidoxime in OP insecticide poisoning. We believe that further trials are required to assess the risk/benefit of oximes and to explore using lower or shorter dosing regimens or different oximes. In all cases oximes should be continued only where there is continuing evidence of usefulness. Our trial provides evidence that routinely following the WHO recommended high-dose pralidoxime regimen in all patients does not improve survival in OP insecticide self-poisoned patients.

## Supporting Information

Text S1 Study protocol.
(0.07 MB DOC)

Text S2 CONSORT checklist.
(0.05 MB DOC)

## Abbreviations

AIC: Akaike's information criterion
CI: confidence interval
GCS: Glasgow coma scale
HR: hazard ratio
IDMC: independent data monitoring committee
IQR: interquartile range
OP: organophosphorus
RCT: randomised controlled trial
