# Supplementary material for: Pralidoxime in Acute Organophosphorus Insecticide Poisoning—A Randomised Controlled Trial
Source: PLoS Med. 2009 Jun 30;6(6):e1000104. doi: 10.1371/journal.pmed.1000104 (PMC2696321; doi:10.1371/journal.pmed.1000104)
Supplement: Text S1 — Study protocol. (0.07 MB DOC) [file pmed.1000104.s001.doc]

**Study protocol (using MRC proforma)**

**1. Trial identifier (RCT #2)**

RCT of pralidoxime in symptomatic organophosphate poisoning.

**2. The need for the trial**

**2.1 What is the problem to be addressed?**

Medical management of acute organophosphate (OP) pesticide poisoning in north central Sri Lanka. Results from the study should be relevant to many other parts of the developing world where such poisoning is common.

**2.2 What are the principal research questions to be addressed?**

Whether pralidoxime will reduce the rate of death and serious complications in patients with symptomatic OP poisoning already receiving atropine. In particular, whether pralidoxime will reduce the requirement for, and length of, admission to an intensive care unit (ICU).

**2.3 Why is a trial needed now?**

Deliberate self-poisoning has reached epidemic proportions in parts of the developing world where highly toxic poisons and sparse medical facilities ensure a high fatality rate.[1,2] Pesticides are the major problem - the WHO estimates that they cause more than 220,000 deaths worldwide each year, most due to OP insecticides.[3] Self-poisoning is particularly important in Sri Lanka where thousands of people die each year and preventing suicide has become a national public health priority.[4] The case fatality rate of OP poisoning in Sri Lanka is ~20% but may reach 60% in some hospitals.[1,2] Better medical management is urgently required.

Standard therapy for OP poisoning involves the reduction of absorption with gastric lavage and/or activated charcoal, followed by atropine and pralidoxime to counter the effect of absorbed pesticide. The use of oximes is controversial. Pralidoxime reactivates inhibited cholinesterases by removing the phosphoryl group left by the pesticide. The compound occurs in two common forms: pralidoxime chloride (2-PAM; used worldwide) and mesylate (P2S; used in the UK).

Clinical experience in Asia has lead to widespread doubt about the relevance of oximes for OP poisoning. A Sri Lankan group found no difference in outcome when pralidoxime was unavailable in their hospital.[5] They argued that pralidoxime is of no clinical benefit and should not be used. Proponents of oximes, including the WHO, have responded that these physicians were using too low a dose and that at least a 30mg/kg loading dose followed by 8mg/kg infusion is required for clinical benefit.[6] Unfortunately, no RCT has thus far been established to test this hypothesis.

**2.4 Give references to any relevant systematic reviews and discuss the need for your trial in the light of these reviews**

There have been no published systematic reviews of pralidoxime in OP poisoning. During 2001, we carried out a systematic review of Medline, Embase, and Cochrane Collaboration databases and discussed the subject with experts in the field, to identify relevant RCTs.[7] Two Indian RCTs have been published.[8,9] Unfortunately, both studies were small and the pralidoxime regimens used not currently recommended (either 1g stat or 12g over 4 days). In particular, while the second study of 100 patients found increased mortality with an infusion of 12g of pralidoxime over 4 days, no loading dose was given and it appears unlikely that a therapeutic plasma concentration was reached. Moreover, mean cholinesterase levels were markedly different at baseline in the two groups, suggesting problems with randomisation and possibly selection of more severely ill patients into the intervention arm. A RCT is required to determine the efficacy of the current WHO-recommended regime of pralidoxime.

**2.5 How will the results of these trials be used?**

We will work with the Sri Lankan Ministry of Health and the WHO’s South East Asia Regional Office (SEARO) from the outset of the study to ensure that its findings are rapidly implemented into clinical practice. The problem is well recognised by SEARO and the Sri Lankan Government and both organisations support the proposed study. We are also collaborating with the International Programme for Chemical Safety’s INTOX program at the WHO to also aid early dissemination of the results.

**3. The proposed trial**

**3.0 Give a brief summary of the proposed trial (<100 words).**

We propose to carry out a double-blind RCT of pralidoxime in adult patients presenting with a history and symptoms of organophosphate poisoning. Primary outcome will be in-hospital mortality; secondary outcomes will include the occurrence of serious complications (respiratory arrest, intermediate syndrome) and time requiring assisted ventilation. Analysis will be on an intention-to-treat basis; the effects of reported time to treatment after poisoning and status on admission will also be assessed.

This RCT will be nested into a study of activated charcoal in unselected cases of poisoning (RCT#1). All investigations and outcome assessments for RCT#1 will suffice for RCT#2. Extra blood samples will not be taken from patients in RCT#2.

**3.1 What is the proposed trial design?**

Randomised double-blind placebo controlled trial with two parallel groups: pralidoxime vs saline.

**3.2 What are the planned trial interventions?**

On recruitment to the study, all patients will receive bolus doses of atropine sufficient to produce ***all of***: heart rate >80/min, mid-sized pupils, normal bowel sounds, and no salivation or bronchorrhoea. At this point, an infusion will be set up to administer 1mg/hr of atropine until clinical improvement (resolution of signs and symptoms) or death. Extra atropine will be given as required to control cholinergic symptoms - this will be recorded.

Patients in the treatment arm will receive a loading bolus of 2g pralidoxime chloride followed by an infusion of 500mg/hour until clinical recovery. It will not be possible to weigh patients rapidly on their admission to hospital. In our previous study, we found that the Sri Lankan poisoning patients had a mean weight of 48.2kg (SD8.9).[9] Therefore, such a regimen will follow the current guidelines for pralidoxime of at least a 30mg/kg bolus dose followed by 8mg/kg/hr infusion.[6; JA Vale, personal communication] Control patients will receive bolus and infusion of saline.

Patients giving consent to enter into RCT#1 will also be randomised to receive a single dose of activated charcoal, multiple doses, or no charcoal. The pralidoxime/ placebo and activated charcoal treatments will be started simultaneously in patients randomised to receive charcoal. Gastric lavage will not be carried out in these studies (see protocol for RCT#1)

A 10ml blood sample will be taken using sterile syringes and needles from each patient before recruitment. Further 5ml blood samples will be taken at one, four and twelve hours post-treatment, and then at daily intervals until discharge or death. Whether a needle or indwelling cannula is used will be decided by the patient. The specific identity and blood concentration of the poison, together with blood acetylcholinesterase activity, will be assayed retrospectively. Population pharmacokinetic analysis of pralidoxime levels will be carried out on 1-3 samples from each patient. Samples of urine and, if possible, the relevant pesticide will be taken on admission for toxicological analysis.

Patients surviving to discharge will have simple psychometric testing performed using Raven’s matrices and the Folstein minimental test.

Throughout the trial, the patient will remain under the responsibility of the admitting consultant. Both study and host hospital clinicians will report to this individual.

**3.2b Consent**

Written informed consent will be requested from conscious patients by a study physician in the patient’s own language. For patients between 14 and 16 years, written informed consent will be obtained from the patient’s parents/guardian; consent will also be sought from these young patients themselves. As requested by the Sri Lankan Ethics committee, consent for unconscious patients will be sought from accompanying relatives. Unaccompanied unconscious patients will not be recruited.

**3.3 What are the practical arrangements for allocating patients to trial groups?**

Participants will be randomised by one of the study team at the bedside using a handheld computer and a specially written programme. Patients will only be randomised once patient data has been entered; after this has been done, patients will not be withdrawn from the study. The doctor will not be able to predict the allocation of any patient before randomisation.

Randomisation will be performed using the method of stratification according to the following factors:

1. reported time between poisoning and recruitment (<4hrs; 4hrs-12hrs; >12hrs; not known);

2. status on admission: asymptomatic

symptomatic with GCS 15/15

symptomatic with GCS <15/15

3. diethyl or dimethyl status of the OP pesticide, if known

4. allocation in RCT #1

**3.4 What are the proposed methods for protecting against other sources of bias?**

All practicable steps have been taken to avoid bias: (i) the randomisation program has been designed to be rapid and simple to operate, and yet remain independent of the investigators; (ii) the next treatment allocation cannot be predicted in advance; (iii) the trial will be double-blind; (iv) the primary outcome, vital status at discharge, is unambiguous, and the secondary outcomes are objective; (v) all outcomes will be recorded by the study team, not other hospital physicians; (vi) patient follow-up will be near 100% complete; and (vii) analysis will be performed on an intention-to-treat basis.

**3.5 What are the planned inclusion/exclusion criteria?**

We hope to recruit all patients admitted to the medical wards with a history of acute poisoning and symptoms and signs of organophosphate poisoning, except for those:

1. under the age of 14 years

2. known to be pregnant

**3.6 What is the proposed duration of treatment?**

Patients will receive an infusion for a maximum of 7 days, or until clinical improvement (resolution of signs and symptoms), or death.

**3.7 What is the proposed frequency and duration of follow up?**

Patients will be seen at least twice a day during their hospital stay, more often as required. Patients will be followed up until hospital discharge.

**3.8 What are the proposed outcome measures?**

1°: all-cause mortality at hospital discharge

2°: % of patients requiring intubation,

time requiring ventilation,

% of patients developing the intermediate syndrome (cranial nerve palsies and/or proximal weakness, without distal weakness, after resolution of the cholinergic crisis).

**3.9 How will the outcome measures be measured at follow-up?**

The main outcome is whether the patient survives to hospital discharge. Respiratory failure requiring intubation will be recorded on CRFs at the time of occurrence. Period of time requiring ventilation will be recorded prospectively - the decision to wean and extubate a patient will be made by the host hospital’s ICU physicians (who will be blinded to pralidoxime therapy).

**3.10 What is the proposed sample size?**

In both hospitals, 20% of OP poisoned patients die before discharge. ~80% of patients become symptomatic; mortality in symptomatic patients is therefore 25%. A reduction (absolute) of 6% would be clinically significant. In order to be able to detect whether pralidoxime reduces the death rate from 25% to 19%, with a significance level (alpha) of 5% and a power of 80%, a minimum of 750 patients must be recruited to each arm of the trial (i.e. 1,500 patients in total).

Sample size calculations are based on current practice and a case fatality rate of 20%. It is possible that part of the reason for the currently high mortality rate is poor control of the airway in these very ill patients. Simple careful control of the airway by the study team may markedly reduce the event rate and compromise the sample size calculations. Therefore, the Data Monitoring and Ethics Committee will be asked specifically to review the recruitment and event rates during the first year of the trial in order to make recommendations about expanding the trial by including further hospitals if necessary (see 3.14).

**3.11 What is the planned recruitment rate?**

Team members will be present in the hospitals at all times - they will be notified of poisoned patients by the doctor on duty in the OPD and go to the medical wards to recruit them. Each hospital sees approximately 360 recruitable patients each year. Past experience of poisoning trials in Sri Lanka suggest a high recruitment rate (>90% in the anti-digoxin Fab RCT [10]); we conservatively presume a 70% recruitment rate. 3 years should be sufficient time to recruit 1,500 patients (see also 3.10, 3.14).

**3.12 Are there likely to be any problems with compliance?**

The intervention will be given by the study team or ward nurses while the patient is under supervision in hospital. We do not foresee any compliance problems.

**3.13 What is the likely rate of loss to follow-up?**

There should be no loss to follow up for the primary outcome. Patients will either be discharged alive from the wards or their bodies transferred to the morgue for judicial autopsy. All secondary outcomes will be assessed in hospital before patient discharge.

**3.14 How many centres will be involved?**

Two to four. Initially Anuradhapura and Polonnaruwa General Hospitals in north central Sri Lanka, with the option of adding in Kandy and Kurunegala General Hospitals as required (see 3.10-3.11).

**3.15 Give details of the planned analyses.**

The RCT will aim to determine whether a loading dose and then infusion of pralidoxime reduces the death rate of symptomatic OP poisoned patients from 25 to 19%.

Main analysis will be carried out on an intention-to-treat basis, using the chi squared test for the primary outcome (or Fisher’s exact test if appropriate) and for other dichotomous outcomes. Relative risk (plus risk reduction), absolute risk reduction and number needed to treat (plus 95% confidence intervals) will also be calculated. For outcomes where time-to-event is recorded, the logrank test will be used to compare treatment groups. In addition, a Kaplan-Meier curve will be produced to illustrate the comparison of mortality between groups.

An analysis of trends in treatment effect for factors ‘reported time from ingestion to treatment’ and ‘patient status on admission’ will also be performed using statistical modelling techniques.

Admission blood samples will be retrospectively analysed to confirm the identity of the poison ingested as an OP and determine its dimethyl/diethyl status. The primary analyses will then be repeated with correction for the identity of the poison and this diethyl/dimethyl status.

**3.15b Hypotheses.**

The main hypothesis is that the pralidoxime will reduce the case fatality rate from 25% to 19%, hence the primary comparison will be pralidoxime vs placebo. The dimethyl vs diethyl state of the OPs is thought to be fundamental for the effectiveness of oximes in OP poisoning. Dimethylated acetylcholinesterase ages quickly such that oximes do not work *in vitro* more than 12hrs post-ingestion; in contrast, diethylated acetylcholin-esterase age slower so that oximes *in vitro* work for 3-4 days post-ingestion. Therefore, once the dimethyl/diethyl status has been retrospectively determined, the analysis will be repeated separating the two groups of OP agents.

It is possible that the oxime, if effective in reducing case fatality rates, will be more effective the earlier it is started. Therefore we will assess the trends in clinical effectiveness according to time post-ingestion to start of therapy. This will be repeated once dimethyl compounds have been distinguished from diethyl compounds.

We also want to determine whether treatment should be started irrespective of severity. We will therefore assess trends in case fatality rates across a gradient of severity.

We hypothesise that pralidoxime treatment will prevent the occurrence of the intermediate syndrome; this will therefore be an important secondary outcome. This analysis will be repeated once retrospective toxicological analysis has separated dimethyl compounds from diethyl compounds.

**3.16 Are there any planned subgroup analyses?**

Subgroup analyses are planned to look at the consistency of treatment effect across different types of pesticide i.e. dimethylated vs diethylated OP pesticides, and for locally common pesticides. A further subgroup analysis will be performed according to the presence of reactivatable acetylcholinesterase before treatment, following retrospective assays of *ex-vivo* reactivatability.

**3.17 What is the proposed frequency of analyses?**

An independent Data Monitoring and Ethics Committee (DMEC) has been established for the trial. For the duration of recruitment, interim analyses will be supplied by the trial statistician (Ed Juszczak), in strict confidence, to the DMEC, together with any other analyses the DMEC may request. The data will be supplied to the Chair of the DMEC as often as he requests. Meetings will be arranged periodically, as considered appropriate by the Chair. In the light of interim data, and other evidence from relevant studies, the DMEC will inform the principal investigator (Dr Michael Eddleston), if in their view (i) there is proof beyond reasonable doubt that the data indicate that any part of the protocol under investigation is clearly indicated or contra-indicated, either for all participants or for a particular subgroup of trial participants, or (ii) it is evident that no clear outcome will be obtained. The decision to inform the principal investigator in either of these circumstances will, in part, be based on statistical considerations.

Appropriate criteria for proof beyond reasonable doubt cannot be specified precisely. A difference of at least three standard deviations in the interim analysis of the major endpoint may be needed to justify halting, or modifying, such a study prematurely. If this criterion were to be adopted, it would have the practical advantage that the exact number of interim analyses would be of little importance, and so no fixed schedule is proposed.[11] Unless modification or cessation of the protocol is recommended by the DMEC, the principal investigator, co-investigators, collaborators and administrative staff will remain ignorant of the results of the interim analyses. Collaborators and all others associated with the study may write to the Chair of the DMEC to draw attention to any concern they may have about the possibility of harm arising from the treatment under study, or any other matter that may be relevant.

**Members of the DMEC:**

Dr Mike Clarke, Cochrane Collaboration, Oxford (Chairman).

Statistician - Dr. Julian Higgins, MRC Biostatistics Unit, Cambridge.

Professor Keith Hawton, Dept Psychiatry, Oxford University.

Professor Saroj Jayasinghe, Dept Medicine, Colombo University (to be confirmed).

Professor Nimal Senanayake, Dept Medicine, Peredeniya University.

Professor Krisantha Weerasuriya, SEARO/WHO, Delhi.

**3.18 Will the trial address any economic issues?**

No. Pralidoxime is currently available in the Sri Lankan Health Services. The issue here is whether it is effective.

**4. Details of trial team**

Dr Michael Eddleston, Oxford. Applicant, principal investigator, trialist in Sri Lanka.

Prof David Warrell, Oxford. Co-applicant, co-investigator; overview in UK /Sri Lanka.

Prof Rezvi Sheriff, Colombo. Co-applicant, co-investigator; overview in Sri Lanka.

Dr Nick Buckley, Canberra. Co-applicant, co-investigator; trialist in Sri Lanka.

Mr Ed Juszczak, Oxford. Co-applicant, co-investigator, study statistician.

Prof Ladislaus Szinicz, Munich. Toxicological analysis in Germany.

Prof Peter Eyer, Munich. Toxicological analysis in Germany.

Prof von Meyer, Munich. Toxicological analysis in Germany.

**5. Further details requested for Lancet Protocol Review**

**Funder:** Tropical Interest Group, The Wellcome Trust.

**Consent form:**  attached

**Information sheet:**  attached

**Ethics:** Approval has been given by the Research Ethics Committee of the Faculty of Medicine, University of Colombo, Sri Lanka, and the Central Oxford Research and Ethics Committee.

**Trial commencement date:** March 2002

**Trial finishing date:**  March 2006

**Trial reporting date:**  March 2007

**Indemnity:** Covered by Oxford University

**References:**

1. Eddleston M, Sheriff MH, Hawton K. Deliberate self harm in Sri Lanka: an over-looked tragedy in the developing world. BMJ 1998; 317:133-5.

2. Eddleston M. Patterns and problems of deliberate self-poisoning in the developing world. QJM 2000; 93:715-31.

3. Jeyaratnam J. Acute pesticide poisoning: a major global health problem. World Health Stat Q 1990; 43:139-44.

4. Presidential Secretariat. Suicide: reducing opportunities and improving treatment. Analyses 1999; 29 June 1999: Number 11.

5. de Silva HJ, Wijewickrema R, Senanayake N. Does pralidoxime affect outcome of management in acute organophosphate poisoning? Lancet 1992; 339: 1136-1138.

6. Johnson MK, Jacobsen D, Meredith TJ, et al. Evaluation of antidotes for poisoning by organophosphorus pesticides. Emergency Medicine 2000; 12:22-37.

7. Eddleston M, Szinicz L, Eyer P, Buckley N. Oximes in acute organophosphate pesticide poisoning. A systematic review of clinical trials. Submitted 2001.

8. Johnson S, Peter JV, Thomas K, Jayaseelan L, Cherian AM. Evaluation of two treatment regimens of pralidoxime (1gm single bolus dose vs 12gm infusion) in the management of organophosphorus poisoning. J Assoc Phys India 1996; 44: 529-31.

9. Cherian AM, Peter JV, Samuel JV, Jaydevan R, Peter S, Joel F, Jaysela F, Thomas K. Effectiveness of 2-PAM (pralidoxime) in the treatment of organophosphorus poisoning, a randomised double-blind placebo-controlled trial. J Assoc Phys India 1997; 45:22-24.

10. Eddleston M, Rajapakse S, Rajakanthan, Jayalath S, Sjostrom L, Santharaj W, Thenabadu P, Sheriff R, Warrell D. Anti-digoxin Fab fragments in cardiotoxicity induced by ingestion of yellow oleander: a randomised controlled trial. Lancet 2000; 355: 967-972.

11. Peto R, Pike MC, Armitage P, et al. Design and analysis of randomised clinical trials requiring prolonged observation of each patient. I. Introduction and design. Br J Cancer 1976; 34:585-612.
